# RETRACTION: Impact of Preoperative Chemotherapy on Cutaneous Wound Healing in Lung Cancer Patients: A Meta‐Analysis

**DOI:** 10.1111/iwj.70477

**Published:** 2025-04-02

## Abstract

**RETRACTION:** ZhaoJ. , CuiH. , QuM. , XuZ. , ZhangY. , and MaC. , "Impact of Preoperative Chemotherapy on Cutaneous Wound Healing in Lung Cancer Patients: A Meta‐Analysis,” International Wound Journal 21, no. 4 (2024): e14518, 10.1111/iwj.14518.38116717 PMC10961031

The above article, published online on 20 December 2023, in Wiley Online Library (http://onlinelibrary.wiley.com/), has been retracted by agreement between the journal Editor in Chief, Professor Keith Harding; and John Wiley & Sons, Ltd. Following an investigation by the publisher, all parties have concluded that this article was accepted solely on the basis of a compromised peer review process. The editors have therefore decided to retract the article. The authors did not respond to our notice regarding the retraction.

**RETRACTION:** J. Zhao , H. Cui , M. Qu , Z. Xu , Y. Zhang , and C. Ma , "Impact of Preoperative Chemotherapy on Cutaneous Wound Healing in Lung Cancer Patients: A Meta‐Analysis,” International Wound Journal 21, no. 4 (2024): e14518, 10.1111/iwj.14518.38116717 PMC10961031

The above article, published online on 20 December 2023, in Wiley Online Library (http://onlinelibrary.wiley.com/), has been retracted by agreement between the journal Editor in Chief, Professor Keith Harding; and John Wiley & Sons, Ltd. Following an investigation by the publisher, all parties have concluded that this article was accepted solely on the basis of a compromised peer review process. The editors have therefore decided to retract the article. The authors did not respond to our notice regarding the retraction.

